# A novel protein truncating mutation in *L2HGDH* causes L-2-hydroxyglutaric aciduria in a consanguineous Pakistani family

**DOI:** 10.1007/s11011-021-00832-2

**Published:** 2021-11-01

**Authors:** Muhammad Muzammal, Muhammad Zeeshan Ali, Beatrice Brugger, Jasmin Blatterer, Safeer Ahmad, Sundas Taj, Syed Khizar Shah, Saadullah Khan, Christian Enzinger, Erwin Petek, Klaus Wagner, Muzammil Ahmad Khan, Christian Windpassinger

**Affiliations:** 1 Gomal Center of Biochemistry and Biotechnology, Gomal University, D.I.Khan, Pakistan; 2 Diagnostic and Research Institute of Human Genetics, Medical University of Graz, 8010 Graz, Austria; 3 Department of Biotechnology and Genetic Engineering, Kohat University of Science and Technology (KUST), Kohat, 26000 Khyber Pakhtunkhwa Pakistan; 4 Department of Neurology, Medical University of Graz, 8010 Graz, Austria

**Keywords:** L-2-hydroxyglutaric aciduria, Whole exome sequencing, Intellectual disability, *L2HGDH*, Leukoaraiosis, N-terminal frameshift mutation

## Abstract

**Background:**

L-2-hydroxyglutaric aciduria (L2HGA) is a rare neurometabolic disorder that occurs due to accumulation of L-2-hydroxyglutaric acid in the cerebrospinal fluid (CSF), plasma and urine. The clinical manifestation of L2HGA includes intellectual disability, cerebellar ataxia, epilepsy, speech problems and macrocephaly.

**Methods:**

In the present study, we ascertained a multigenerational consanguineous Pakistani family with 5 affected individuals. Clinical studies were performed through biochemical tests and brain CT scan. Locus mapping was carried out through genome-wide SNP genotyping, whole exome sequencing and Sanger sequencing. For in silico studies protein structural modeling and docking was done using I-TASSER, Cluspro and AutoDock VINA tools.

**Results:**

Affected individuals presented with cognitive impairment, gait disturbance, speech difficulties and psychomotor delay. Radiologic analysis of a male patient revealed leukoaraiosis with hypoattenuation of cerebral white matter, suggestive of hypomyelination. Homozygosity mapping in this family revealed a linkage region on chromosome 14 between markers rs2039791 and rs781354. Subsequent whole exome analysis identified a novel frameshift mutation NM_024884.3:c.180delG, p.(Ala62Profs*24) in the second exon of *L2HGDH*. Sanger sequencing confirmed segregation of this mutation with the disease phenotype. The identification of the most N-terminal loss of function mutation published thus far further expands the mutational spectrum of *L2HGDH.*

**Supplementary Information:**

The online version contains supplementary material available at 10.1007/s11011-021-00832-2.

## Introduction

L-2-hydroxyglutaric aciduria [L2HGA (OMIM #236,792)] is a rare autosomal recessive neurodegenerative metabolic disorder, that occurs due to accumulation of L-2-hydroxyglutaric acid in the cerebrospinal fluid (CSF), plasma and urine (Duran et al. 1980; Chen et al. 1996). Phenotypic features of the affected individuals are variable and may include developmental delay, moderate to severe intellectual disability, epilepsy, behavioral problems, spasticity, macrocephaly, speech disorders and cerebellar ataxia (Barth et al.^1992^; Barth et al. 1998; Hanefeld et al.^1994^). Age of onset of L2HGA (OMIM #236,792) is variable and may occur at an early age with severe epilepsy and intellectual disability or in adulthood with moderate to mild symptoms. Different studies have documented that an elevated level of L2HGA (OMIM #236,792) in the brain may also lead to brain tumors (Steenweg et al. 2010; Haliloglu et al. 2008). Diagnosis of L2HGA can be established by means of radiological, biochemical and genetic testing. Metabolic screening includes plasma amino acid and urine organic acid analysis. Radiological examinations such as MRI and CT are necessary for the detection of brain abnormalities, especially with regard to subcortical cerebral white matter, globus pallidus, putamen, caudatus and dentatus, which are specifically affected by L2HGA (Moroni et al.^2004^; Seijo-Martínez et al. 2005; Topcu et al. 2005).

*L2HGDH* is expressed in various tissues with highest expression in brain, followed by muscles and testis (Vilarinho et al. 2005). The corresponding protein consists of 463 amino acids, which contains two domains i.e. a mitochondrial targeting sequence and a FAD dependent oxidoreductase domain (UniProtKB: Q9H9P8) (Goffette et al. 2006). *L2HGDH* acts as a mitochondrial enzyme which is involved in glutamate and glutamine metabolism pathways. Its prime function is to catalyze the oxidation of L-2-hydroxyglutarate (L2HG) to α2-ketoglutarate (α2KG) (Topçu et al. 2004; Vilarinho et al. 2009). Exact prevalence of L2HGA (OMIM #236,792) is unknown, but approximately 140 cases have been reported to date (Goffette et al. 2006; Topçu et al. 2004; Vilarinho et al. 2009; Jellouli et al. 2014; Larnaout et al. 2008; O'Connor et al.^2009^). Although there is no established treatment of L2HGA, Samuraki et al. reported effective treatment of a late onset patient with flavin adenine dinucleotide sodium (FAD) and levocarnitine chloride (Samuraki et al. 2008).

In the present study, we report on a consanguineous Pakistani family displaying mild intellectual disability. Genome-wide homozygosity mapping coupled with whole exome sequencing revealed a novel frameshift mutation NM_024884.3:c.180delG, p.(Ala62Profs*24) in the 2^nd^ exon of *L2HGDH*. The identified mutation presumably creates a premature stop codon ether leading to nonsense mediated mRNA decay or truncation of the protein, which would distort the local folding of the polypeptide chain and lead to loss of its interacting sites.

## Methods

The current study was approved by the institutional ethical review board of Gomal University, Dera Ismail Khan, Pakistan, and patients were enrolled after obtaining written informed consent. The family was ascertained from Dera Ismail Khan, City in Khyber Pakhtunkhwa province of Pakistan. Blood samples were taken from available affected (V:4, V:5 and V:8) and unaffected (IV:2 and V:6) family members. DNA was extracted using standard laboratory protocols.

## Clinical assessment

The clinical assessment of patients was carried out through biochemical tests e.g. liver functioning test (LFTs), renal function tests (RFTs), urine organic acid analysis and plasma amino acid analysis. Radiologic analysis was performed through CT scan of affected individual V:8.

## Genome-wide SNP genotyping

Whole genome SNP genotyping was performed through microarray analysis using the Infinium Global Screening Array (Illumina, USA) kit. Raw data analysis was performed at the Life and Brain GmbH, Bonn, Germany. Homozygosity mapping to identify the disease associated locus, was carried out using GenomeStudio 2.0 Software (Illumina, USA).

## Whole exome sequencing (WES)

For genetic analysis whole exome sequencing (WES) was performed for a single affected individual (V:4) via Agilent SureSelect V6 human All Exon library preparation, sequencing was conducted using a NovaSeq 6000 with 2 × 150 bp and 100 × coverage (50 × on-target coverage). Sequence alignment of raw fastq files to the human reference sequence (GRCh37/hg19 assembly) and variant calling was performed with the DRAGEN Germline Pipeline 3.2.8 on Illumina BaseSpace (https://basespace.illumina.com/). Variant annotation, analysis and homozygosity mapping was performed using VarSeq™ v2.2 (Golden Helix, Inc., Bozeman, MT, www.goldenhelix.com).

## Segregation analysis

For Sanger DNA sequencing, primers were design through Primer3web tool (version 4.1.0) (Untergasser et al. 2012). Sanger sequencing was performed for available study participants (IV:2, V4-V8). Sequence analysis was performed by online BLAT tool package available in UCSC Genome Browser (Sanger et al.^1977^; Kent 2002) and offline BioEdit tool (version 7.0.5).

## In silico studies

Computational analysis of mutant L2HGDH involved protein structural modeling and protein interaction capability.

## Protein structure prediction

Structural modeling was done on I-TASSER tool (Yang and Zhang 2015) and models with highest confidence score (C- score) were selected for onward interaction studies. To further confirm the efficiency of predicted structure, I-TASSER (Yang and Zhang 2015) results were also crossed checked by SWISS MODEL (Bienert et al. 2016).

## Molecular Docking and visualization

For interaction studies, ClusPro (Kozakov et al. 2017) tool was used for protein–protein docking between L2HGDH (wildtype and mutated protein) with its close interacting protein, which was predicted through STRING database (Szklarczyk et al. 2018). Protein-substrate docking was performed through AutoDock VINA tool (vina.scripps.edu) (Trott and Olson 2009). and molecular visualization through different offline tools e.g. LigPlot + (Version 2.1)( Laskowski et al.2011), PyMOL 2.3 (Schiffrin et al. 2020), Chimera 1.13.1(Pettersen et al. 2004) and Discovery Studio 2020(https://www.3dsbiovia.com/products/datasheets/discovery-studio-visualizer).

## Results

**Fig. 1:**
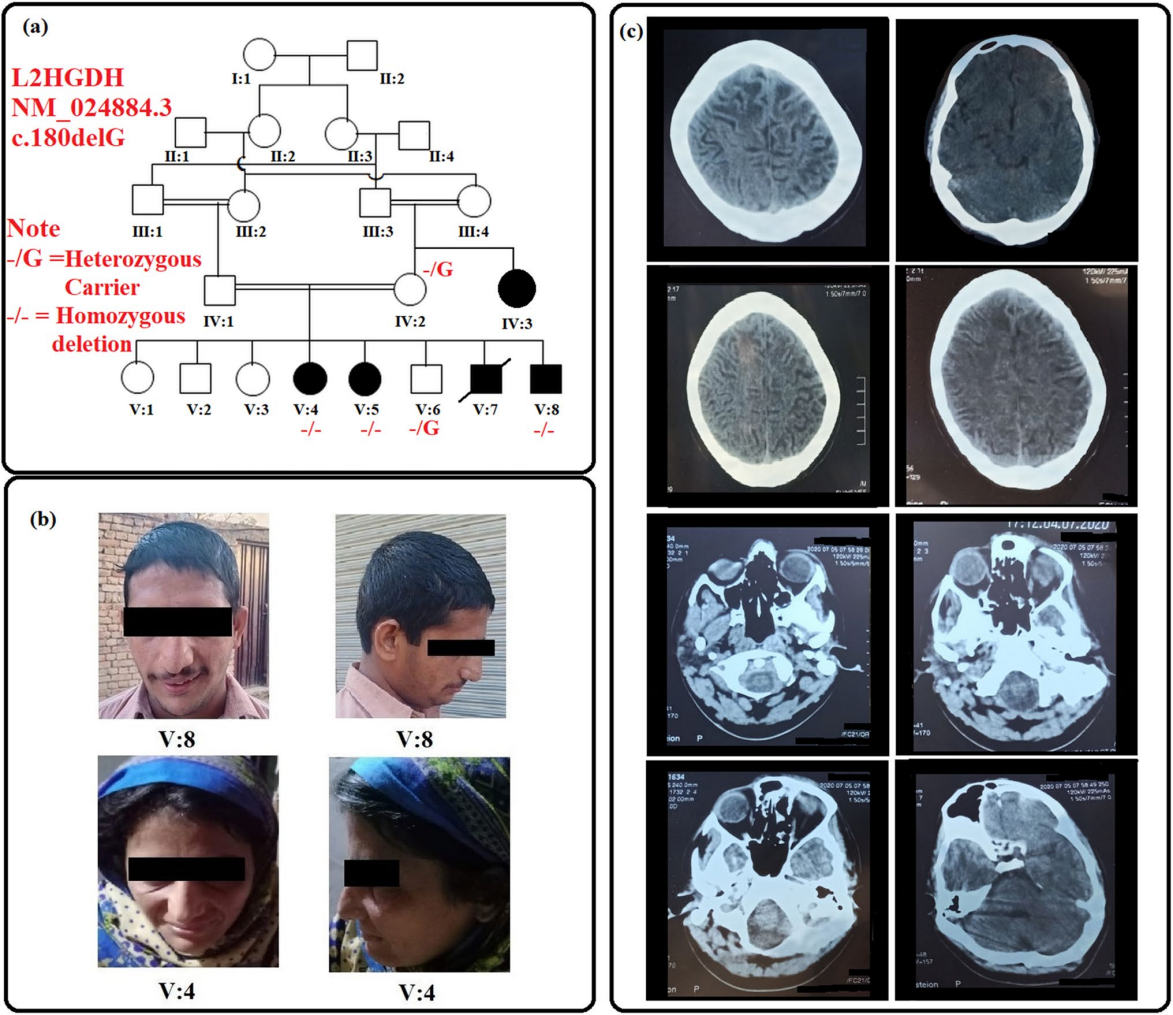
(**a**) Pedigree analysis illustrate autosomal recessive mode of disease segregation, and the genotype status of each analyzed individuals are represented as -/- (homozygous deletion) and -/G (heterozygous carrier). (**b**) Patient's photographs and (**c**) multiple panels of brain CT images of patients V:8 show white-matter atrophy

The current study describes a consanguineous Pakistani family displaying intellectual disability with gait and speech problems recruited from Dera Ismail Khan City in Khyber Pakhtunkhwa province of Pakistan. The family pedigree and clinical history was assessed for five generations, with 4 affected individuals in the 5^th^ generation and a single affected individual in the 4^th^ generation (Fig. 1a).

Molecular genetic analysis revealed a novel frameshift mutation in *L2HGDH* associated with L-2-hydroxyglutaric aciduria.

### Clinical Findings

#### Phenotype 

All affected family members showed mild intellectual disability and developing speech with weak communication skills. The patients had gait disturbance, however, no muscular dystrophy or skeletal anomalies were observed. No digital anomalies were determined, except syndactyly of feet in patient V:8. The affected individuals did not have a feeling of satiety even after excessive eating. Patient V:5 and V:8 had a history of epilepsy during the childhood. The head circumference in all patients was within normal range. Nonetheless, some degree of facial dysmorphism was observed due to drooping mouth (Fig. 1b). The general physique of the patients was normal. Examination of hearing, vision, visceral organs and skin was found normal (a summary of the phenotypic data is given in Table 1).

**Table 1:** Clinical description of patients suffering from L-2-hydroxyglutaric aciduria

Phenotypes	Patient IDs		
	**V:4**	**V:5**	**V:8**
Gender	Female	Female	Male
Age (Years)	23	22	17
Age of Disease onset	Congenital	Congenital	Congenital
General Physique	Normal	Normal	Normal
IQ level	Mild	Mild	Mild
Intellectual disability	Yes	Yes	Yes
Psychomotor Retardation	Yes	Yes	Yes
Behavioral Expression	Normal	Normal	Normal
Level of communication	Weak	Weak	Weak
Level of speech	Developing	Developing	Developing
Epilepsy	No	Yes	Yes
Occipital-head circumference	Normal	Normal	Normal
Facial Dysmorphism	Yes	Yes	Yes
Syndactyly	No	No	Yes (Only in feet)
Polydactyly	No	No	No
Ambulation	Delayed	Delayed	Delayed
Gait	Abnormal	Abnormal	Abnormal
Movement of joints	Normal	Normal	Normal
Morphology of long bones	Normal	Normal	Normal
Muscular Dystrophy	No	No	No
Hearing ability	Normal	Normal	Normal
Dentition	Normal	Normal	Normal
Ophthalmic screening	Normal	Normal	Normal
Visceral organ defect	Not apparent till last visit	Not apparent till last visit	Not apparent till last visit
Dermal, hair and nail examination	Normal	Normal	Normal

#### Radiological Findings

A CT scan was performed for a male patient (V:8). Plain CT demonstrates leukoaraiosis with hypoattenuation of cerebral white matter, particularly evident in frontal lobes. Also, a left-hemispheric preponderance becomes apparent (also involving capsula externa). The gyration appears normal, there is slight widening of the lateral ventricles, but no evident atrophy pattern in this 18-year-old male (Fig. 1c).

#### Biochemical Findings

Serum biochemistry reports showed high serum creatinine levels, however, blood urea, bilirubin, alanine transaminase (ALT), and alkaline phosphatase levels were normal. Urine organic acid analysis revealed marked excretion of 2-hydroxglutaric acid with a small peak for glutaric acid. A peak for 2-hydroxyglutaric lactones was not identified. Additionally, plasma amino acid analysis exhibited nonspecific variations in the level of different amino acids. For example, level of glutamate, glycine, alanine, leucine, ornithine and lysine were abnormally high, while, value of the cysteine was below the reference range. Patient’s biochemistry profile is illustrated in Table 2.

**Table 2:** Biochemical profile of patients suffering from L-2-hydroxyglutaric aciduria

S No	Test		Normal Range	Patient’s Result
1	Renal Functioning test (RFTs)	Blood Urea	10–50 mg/dl	47
		Serum creatinine	0.6–1.4 (adult) mg/dl	1.7
		Bilirubin	0.1–1.2 mg/dl	1.1
2	Liver Functioning test (LFTs)	Alt/SGPT	09–45(Male) U/l	37
		ALK. Phosphatase	Up to -303 U/l	290
3	Metabolic Screening	Urine Organic Acid		Marked excretion of 2-hydroxyglutaric acid
		Below normal range amino acids		
		Cysteine	32–64	15 umol/L
		Border line high range amino acids		
		Valine	142–278	282 umol/L
		Isoleucine	38–94	104 umol/L
		Histidine	58–106	125 umol/L
		Aspartate	4–28	41 umol/L
		Serine	75–175	183 umol/L
		Asparagine	32–64	76 umol/L
		Phenylalanine	38–78	106 umol/L
		Taurine	10–162	187 umol/L
		Abnormally high amino acids		
		Glutamate	11–59	225 umol/L
		Glycine	148–324	406 umol/L
		Alanine	192–508	779 umol/L
		Leucine	76–168	250 umol/L
		Ornithine	20–84	202 umol/L
		Lysine	105–221	414 umol/L
		Normal range amino acids		
		Threonine	72–192	175 umol/L
		Glutamine	396–740	429 umol/L
		Citrulline	17–49	30 umol/L
		Methionine	16–36	26 umol/L
		Tyrosine	40–92	87 umol/L
		Arginine	45–125	45 umol/L
		Proline	75–307	291 umol/L

**Note:** Clinical laboratory tests were performed only on patient V:8 due to unavailability and non-cooperativeness of other patients

### Molecular Findings

**Fig. 2:**
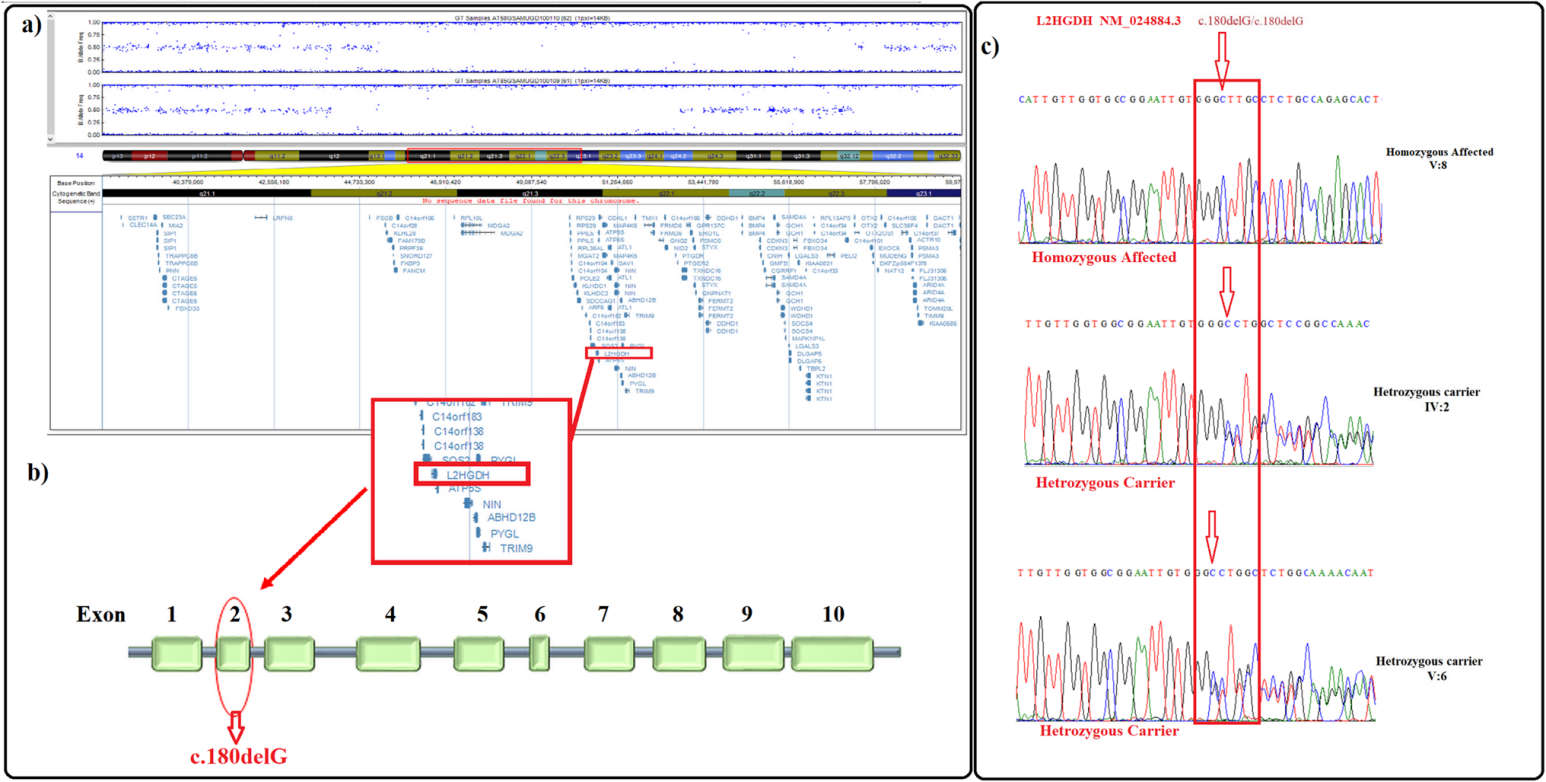
Panel (**a**) shows SNP genotyping based HBD region and list of candidate genes between markers rs2039791 and rs781354. The causative gene *L2HGDH* is enclosed in red box (**b**) The structure of L2HGDH gene in which mutation harboring exon is encircled in red (**c**) Sanger sequencing chromatogram shows homozygous deletion in affected (V:8), which normal individuals (IV:2 & V:6) exhibited characteristic heterozygous/ carrier chromatogram. The position of deletion is framed in red box on the chromatogram

Whole genome homozygosity scan revealed a common linkage region on the q arm of chromosome 14 between SNP markers rs2039791 to rs781354 (45,171,670 bp – 52,879,326 bp). The size of the identified linkage interval spans over 7.7 Mb, which harbors 65 protein-coding genes (Fig. 2a).

Whole exome data analysis identified a novel homozygous frameshift mutation NM_024884.3:c.180delG, p.(Ala62Profs*24) in the second exon of *L2HGDH*. Co-segregation of the identified mutation with the disease was confirmed by Sanger sequencing (Fig. 2c). This novel frameshift mutation was not listed in ClinVar, HGMD and gnomAD databases.

### Structural Findings

#### Molecular modeling of Normal and Mutated L2HGDH proteins

**Fig. 3:**
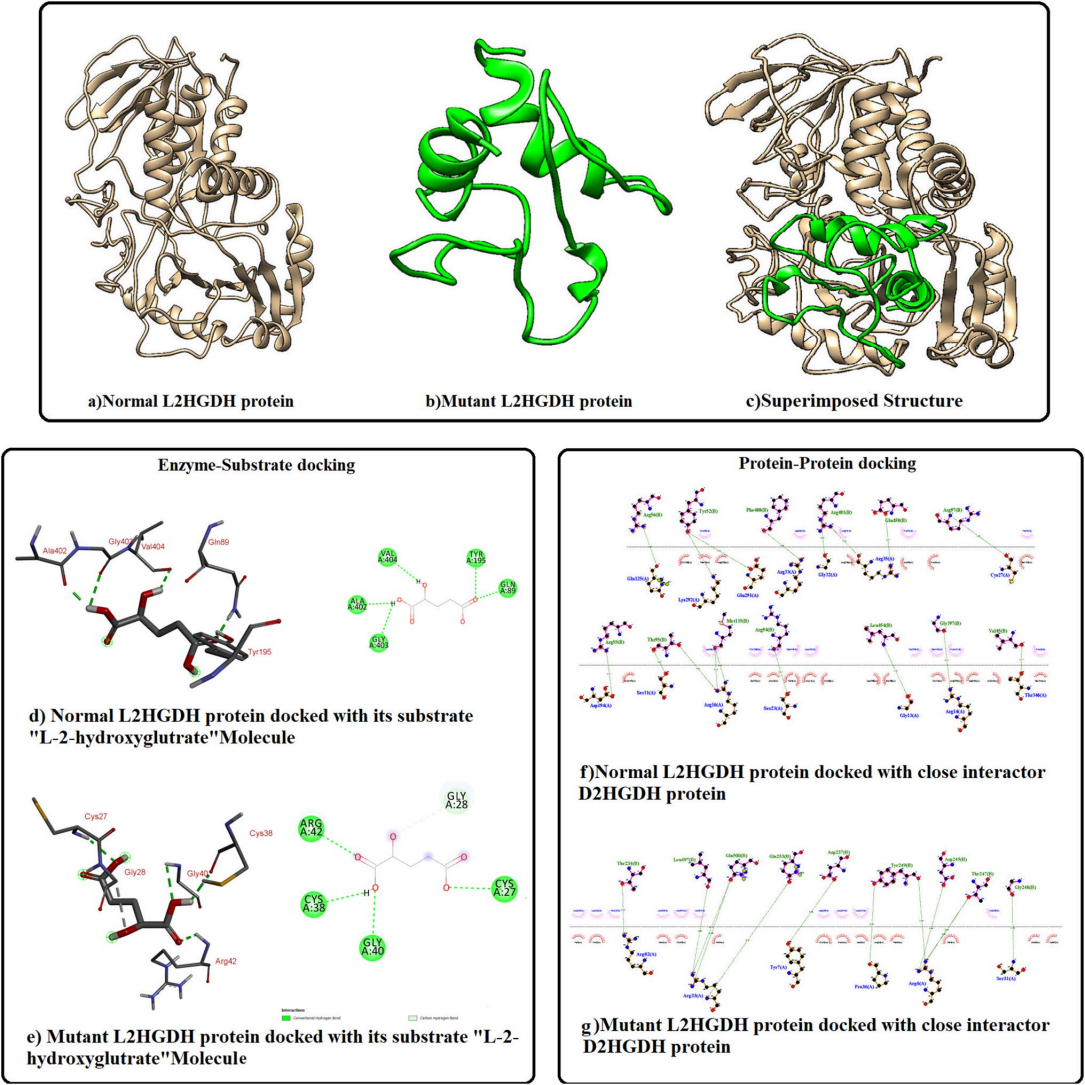
**(a**)Normal L2HGDH protein model (**b**)Mutant L2HGDH protein model (**c**)Superimposed structure (**d**)Normal L2HGDH protein docked to its substrate “L-2-hydroxyglutrate” molecule (**e**)Mutant L2HGDH protein docked to its substrate “L-2-hydroxyglutrate” molecule (**f**)Normal L2HGDH protein docked to its close interactor D2HGDH protein (**g**) Mutant L2HGDH protein docked to its close interactor D2HGDH protein

After doing molecular modeling, the 3D-structures of both wild type and mutated L2HGDH were superimposed, which failed to overlap due to misfolding. It confirms that identified frameshift mutation results in structural distortion of L2HGDH (Fig. 3).

#### Protein–Protein Docking

Interaction studies of L2HGDH with D2HGDH have revealed remarkable alteration in docking sites (see Fig. 3). In addition to this, docking of L2HGDH with other close interactors i.e. ALDH4A1 and GLS2 proteins have exhibited significant alteration in the interacting sites (supplementary figure).

#### Enzyme–Substrate Docking

Interaction studies were also performed for L2HGDH proteins and its substrate i.e. L-2-hydroxyglutarate, which predicted five amino acids i.e. Gln-89, Tyr-195, Val-404, Ala-402 and Gly-403 of wild-type L2HGDH to be involved in interaction with its substrate via conventional hydrogen bonding. All these binding sites are within the FAD dependent enzyme domain. In mutated L2HGDH interacting sites within the FAD domain are lost due to frameshift and protein truncation. However, the mutant enzyme predictably showed interaction with its substrate on different positions i.e. Arg-42, Cys-38, Gly-40 and Cys-27 through conventional hydrogen bond, and Gly-28 through carbon hydrogen bond (see Fig. 3).

## Discussion

L-2-hydroxyglutaric aciduria is a rare form of autosomal recessive neuro-metabolic disorder that is caused by mutations in *L2HGDH*. The corresponding protein acts as a mitochondrial enzyme which bio-oxidizes the L-2-hydroxyglutaric acid to α-ketoglutarate (Olgac et al. 2019), and is involved in butanoate metabolism, glutamate and glutamine metabolism pathways (Olgac et al. 2019; Ma et al. 2017). There are two defined features, the mitochondrial targeting sequence and a FAD dependent oxidoreductase domain (UniProtKB: Q9H9P8). Insufficient enzyme activity leads to toxic levels of L-2-hydroxyglutaric acid in the cerebrospinal fluid (CSF), plasma and urine. The main phenotypical features associated with L2HGA include leukodystrophy, intellectual disability, psycho-motor abnormalities, macrocephaly, intention tremors, abnormal gait, epilepsy and cerebellar atrophy (Haliloglu et al. 2008).

Penderis et al. (2007) described a spontaneous canine model of L-2-hydroxyglutaric aciduria in outbred bull terriers dogs. All affected dogs exhibited increased urinary excretion of L-2-hydroxyglutarate (L-2-HG), while 12 dogs in which MRI imaging was performed showed symmetric regions of hyper intensity comparable to that seen in humans (Penderis et al. 2007).

Similarly, Ma et al. (2017) developed an L2hgdh null mice and found range of phenotypes i.e. increased level of L-2-hydroxyglutarate (L-2-HG) levels in multiple tissues, especially in the brain and testis. L2hgdh null mice demonstrated white matter deterioration, extensive gliosis, microglia-mediated neuro-inflammation, and an expansion of oligodendrocyte progenitor cells. Additionally, L2HGDH deficiency in the later stages results in hippocampal neurogenesis and late-onset neurodegeneration (Ma et al. 2017). Oldham and coworkers identified L-2-hydroxyglutarate (L2HG) as an important factor for the hypoxia response. Earlier, L2HG was reported to be produced by the malate dehydrogenase via mitochondrial 2-oxoglutarate reduction. Elevated level of 2-oxoglutarate is considered responsible for accumulation of L2HG, which happens due to dysfunction of tricarboxylic acid cycle and increased mitochondrial reducing potential. These changes were associated with homeostasis of cellular redox, because elevated level of L2HG in cell prevents glycolysis as well as electron transport, in order to counterbalance the unfavorable consequences of mitochondrial reductive stress provoked by hypoxia. Therefore, L2HG combines cytoplasmic and mitochondrial based energy metabolism in a new cellular redox regulation model (Oldham et al. 2015). Qiu et al. (2020) reported that both mitochondrial enzyme i.e. L2HGDH and D2HGDH catalyzes the oxidation of L2HG and D2HG into α-ketoglutarate. The studies have shown that MYC is the essential factor that regulates the expression of both L2HGDH and D2HGDH. It basically regulates the TET DNA hydroxylases and RNA demethylases, and thereby controls the cellular epigenome and epitranscriptome (Qiu et al. 2020). In addition Ye et al. (2018) have demonstrated the role of 2-HG other than epigenetic control and linked the expression of 2-HG (D-and L-2-Hydroxyglutarates) to T cell regulation and suggest its presumable role in tumor immunity (Ye et al.^2018^).

To date, 83 mutations in *L2HGDH* have been published (according to HGMD, Feb. 2021), however, only two mutations i.e. c.1003C > T p.(Arg335*) (Sass et al. 2008) and c.178G > A p.(Gly60Arg) (Ullah et al.^2018^) have been described in Pakistani families. In this study, we are reporting on a multigenerational Pakistani family presenting with mild intellectual disability, psychomotor retardation, gait disturbance and epilepsy. Whole exome sequencing identified a frameshift mutation NM_024884.3: c.180delG, p.(Ala62Profs*24) in *L2HGDH,* the most N-terminal loss of function mutation in this gene published thus far. The synopsis of the molecular findings and the clinical presentation of patients, based on the biochemical profile and brain CT findings is in concordance with the diagnosis of L2HGA. Subsequent structural and interaction analysis were conducted to predict the functional impact of the mutation in case of protein truncation. Analysis revealed remarkable changes in the local folding of L2HGDH and interaction with its substrate (L-2-hydroxyglutarate) and close interactors (D2HGDH, ALDH4A1, GLS2). However, as the mutation is located in close proximity to the N-terminus, nonsense mediated mRNA decay cannot be ruled out as the underlying patho-mechanism in this family. Some biochemical studies have shown that 2-hydroxyglutaric aciduria may be associated with elevated levels of lysine (Samuraki et al. 2008). Interestingly, biochemical profiling of one of our patients showed additional abnormally high levels of glutamate, glycine, alanine, leucine and ornithine amino acids, but it remains unclear whether these findings can be attributed to the mutation in *L2HGDH*. The comparative clinical analysis of the present family with previously reported Pakistani L2HGA family determined partial overlap (Ullah et al. 2018). However, tonic–clonic seizure and macrocephaly was not present in the patients presented here. Further, Peng et al. (2018) have reported a few missense and frameshift mutations in Chinese patients, who exhibited mild phenotypes comparable to the patients included in the current study (Peng et al. 2018). Additionally, none of the patients do exhibit cerebral neoplasms thus far.

Based on the findings of the current study, it is suggested that pediatricians in developing countries (especially in Pakistan) should offer screening of metabolic disorders in children, because early diagnosis and therapeutic interventions may effectively reduce the progression of the disease.

## Conclusion

Herein, we report on the most N-terminal loss-of-function mutation in *L2HGDH* [NM_024884.3: c.180delG p.(Ala62Profs*24)] in a consanguineous family causing L-2-hydroxyglutaric aciduria. This finding further expands the mutational spectrum of L2HGDH.

## Supplementary Information

Supplementary figure (TIF 1.53 mb)

## Data Availability

The reference sequence data was obtained from UCSC genome browser (http://genome.ucsc.edu/). The patient’s data (sequence, photographs, pedigrees) is stored in the password protected computer of Lab of Medical Genetics at Gomal University, D.I.Khan and is available upon request.
