# Supplementary figures and images for: A novel protein truncating mutation in L2HGDH causes L-2-hydroxyglutaric aciduria in a consanguineous Pakistani family
Source: Metab Brain Dis. 2021 Nov 1;37(1):243–52. doi: 10.1007/s11011-021-00832-2 (PMC8748340; doi:10.1007/s11011-021-00832-2)

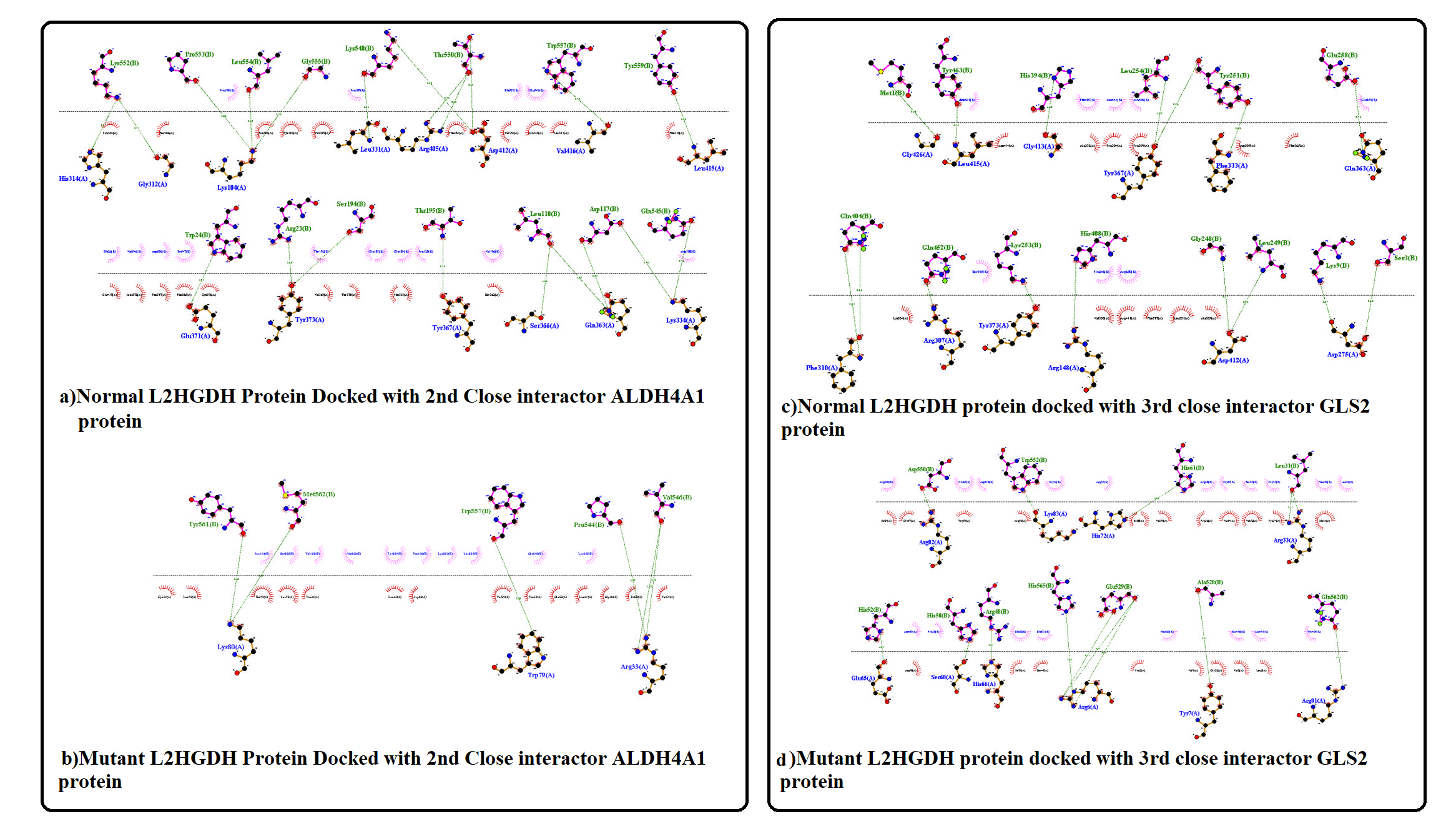

Supplement: Supplementary file 1 — Supplementary figure (TIF 1.53 mb) [file 11011_2021_832_MOESM1_ESM.tif]
